# The complete chloroplast genome of *Azara serrata* (Salicaceae) from Chile

**DOI:** 10.1080/23802359.2018.1535845

**Published:** 2018-11-21

**Authors:** Min Su, Minghui Kang, Mengmeng Li, Deyan Wang, Xiaoting Xu

**Affiliations:** Key Laboratory of Bio-Resource and Eco-Environment of Ministry of Education, College of Life Sciences, Sichuan University, Chengdu, China

**Keywords:** *Azara serrate*, chloroplast genome, phylogenetic analysis

## Abstract

The chloroplast (cp) genome sequence of *Azara serrata* has been characterized from Illumina pair-end sequencing. The complete cp genome was 158,306 bp in length, containing a large single copy region (LSC) of 85,059 bp and a small single copy region (SSC) of 17,889 bp, which were separated by a pair of 27,679 bp inverted repeat regions (IRs). The genome contained 129 genes, including 85 protein-coding genes, 36 tRNA genes, and 8 rRNA genes. The overall GC content is 36.5%, while the corresponding values of the LSC, SSC, and IR regions are 34.3, 30.0, and 42.0%, respectively. Further, phylogenetic analysis suggested that the *A. serrata* is a sister of *Flacourtia indica* and is an outgroup to the remaining genera of Saliaceae.

*Azara serrata*, with common name saw-toothed azara, is an evergreen shrub species as high as 4 m with glossy serrated leaves and native to Chile. This species blooms in summer with fragrant yellow flowers in clusters or spikes from leaf axils. Because of its and high resistance to pest and disease, it was rewarded as Garden Merit by Royal horticultural Society. In addition, the phylogenetic position of *Azara serrata* and the genus *Azara* is still unresolved. In this study, we first reported the complete chloroplast genome of *A. serrata*.

The total genomic DNA was extracted from dry leaves using a modified CTAB method (Doyle and Doyle 1987) and sequenced based on the Illumina pair-end technology. The voucher specimen was collected at region IX, province Melluco, Chile (37.786S, 72.827W) and stored at Gray Herbarium of Harvard University (E.J. Tepe 2162). The filtered reads were assembled using the program NOVOPlasty (Dierckxsens et al. 2017) with complete chloroplast genome of its close relative *Flacourtia indica* as the reference (GenBank accession no. MG262341). The assembled chloroplast genome was annotated using Plann (Huang and Cronk 2015), and the annotation was corrected using Geneious (Kearse et al. 2012). The physical map of the new chloroplast genome was generated using OGDRAW (Lohse et al. 2013). The accurate new annotated complete chloroplast genome was submitted to GenBank with accession number MH719101. The complete chloroplast genome of *A. serrata* is 158,306 base pairs (bp) in length, containing a large single-copy (LSC) region of 85,059 bp, a small single-copy (SSC) region of 17,889 bp, and two inverted repeat (IR) regions of 27,679 bp. The new sequence possesses total 129 genes, including 85 protein-coding genes, 8 rRNA genes, and 36 tRNA genes. Among all of these genes, four rRNA genes (i.e. 4.5S, 5S, 16S, and 23S rRNA), eight protein-coding genes (i.e. ndhB, rpl2, rpl23, rps12, rps19, rps7, ycf15, and ycf2), and seven tRNA genes (i.e. trnA-UGC, trnI-CAU, trnI-GAU, trnL-CAA, trnN-GUU, trnR-ACG, and trnV-GAC) occur in double copies. The overall GC-content of the whole plastome is 36.5%, while the corresponding values of the LSC, SSC, and IR regions are 34.3, 30.0, and 42.0%, respectively.

To further investigate its phylogenetic position, a neighbor-joining tree (Saitou and Nei 1987) was constructed based on complete chloroplast genome sequences of 16 other Saliaceae species using MEGA7 (Kumar et al. 2016) with 1000 bootstrap replicates. Here, we aligned all 16 sequences using MAFFT (Katoh and Standley 2013). Our results showed that *A. serrata* is out of the *Itoa orientalis* and is an outgroup to the genera of *Salix* and *Populus* with over 90% bootstrap support (Figure 1). The phylogenetic position of other species consists with previous study (Zhang et al. 2018).

**Figure 1.**
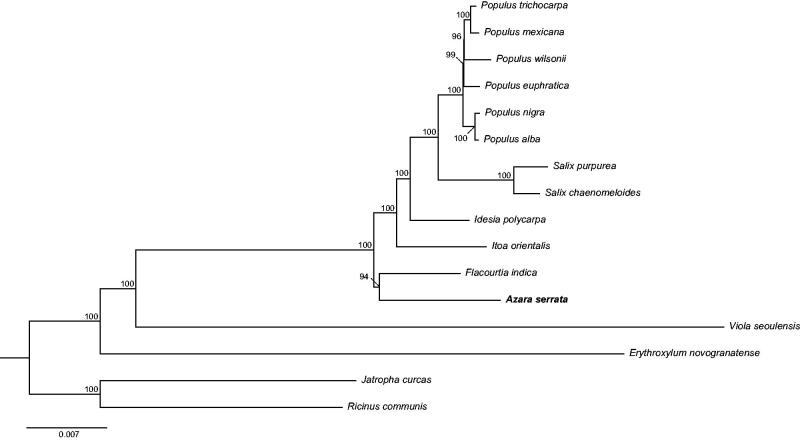
Phylogenetic relationships of 16 Salicaceae species based on chloroplast genome sequences. Bootstrap support is indicated for each branch. GenBank accession numbers: *Erythroxylum novogranatense* (KX256287), *Flacourtia indica* (MG262341), *Idesia polycarpa* (KX229742), *Itoa orientalis* (MG262342), *Jatropha curcas* (FJ695500), *Populus mexicana* (MG262353), *P. alba* (AP008956), *P. euphratica* (KJ624919), *P. nigra* (MG262354), *P. trichocarpa* (EF489041), *P. wilsonii* (MG262359), *Ricinus communis* (JF937588), *Salix chaenomeloides* (MG262362), *S. purpurea* (KP019639), and *Viola seoulensis* (Unpublished).
